# First record of a case of cutaneous loxoscelism caused by violin spider (*Loxosceles yucatana*) bite in Yucatan, Mexico

**DOI:** 10.17843/rpmesp.2022.394.11047

**Published:** 2022-12-05

**Authors:** Daly Martínez-Ortiz, Marco Torres-Castro, Carlos Arisqueta-Chablé, Beatriz Salceda-Sánchez, Herón Huerta, Jorge Palacio-Vargas, Wilbert Bibiano-Marín, Norma Pavía-Ruz, Pablo Manrique-Saide

**Affiliations:** 1 Healthcare Services of Yucatán, Mérida, Mexico. Healthcare Services of Yucatán Mérida Mexico; 2 “Dr. Hideyo Noguchi” Regional Research Center, Universidad Autónoma de Yucatan, Merida, Mexico. Universidad Autónoma de Yucatan “Dr. Hideyo Noguchi” Regional Research Center Universidad Autónoma de Yucatan Mérida Mexico; 3 Collaborative Unit for Entomological Bioassays, Campus of Biological and Agricultural Sciences, Universidad Autónoma de Yucatán, Merida, Mexico. Universidad Autónoma de Yucatán Collaborative Unit for Entomological Bioassays Campus of Biological and Agricultural Sciences Universidad Autónoma de Yucatán Mexico; 4 Laboratorio de Entomología, Instituto de Diagnóstico y Referencia Epidemiológicos, Secretaría de Salud, Ciudad de México, México. Laboratorio de Entomología Instituto de Diagnóstico y Referencia Epidemiológicos, Secretaría de Salud Ciudad de Mexico Mexico

**Keywords:** Cutaneous loxoscelism, *Loxosceles yucatana*, clinical case, Yucatan, Mexico, spiders.

## Abstract

Loxoscelism occurs when the dermonecrotic venom produced by spiders of the genus *Loxosceles*, known as “violin spiders,” enters a person’s organism through their bite. In Mexico there is an underreporting of loxoscelism cases due to the absence of laboratory tests for its diagnosis and the complexity of the clinical picture. The aim of this paper is to describe a case of cutaneous loxoscelism caused by the bite of *Loxosceles yucatana* in a resident of Yucatan, Mexico. Cutaneous loxoscelism is the most frequent and less severe type. This case was diagnosed by means of the symptomatology registered in the medical records, the initial lesion, and the identification of *L. yucatana* spiders. This study represents the first description of a case of cutaneous loxoscelism with favorable outcome in Yucatan.

## INTRODUCTION

Spiders of the genus *Loxosceles* (*L*.) belong to the family Sicariidae, suborder Araneomorphae, order Araneae. A total of 139 species are known worldwide^ (^ ^1^, of which 40 species have been described in Mexico (38 native and two introduced: *L. reclusa* and *L. rufescens*) ^2^ ^,^ ^3^. Only *L. yucatana *has been reported in the State of Yucatan, being in fact, the first species of *Loxosceles* reported in the country, based on specimens collected in caves of this State ^3^.

The term *Loxosceles* (*loxos*: curved and *kelos*: legs) refers to the slightly laterigrade position of their resting legs, which gives them a circular or curved appearance. They are commonly known as “brown spiders,” “fiddle-back spiders,” or “violin spiders” because most species have a characteristic dark spot on the cephalothorax in the shape of a violin with the handle towards its rear end ^3^ ^,^ ^4^. These spiders have a length 8-13 mm including the legs. They are brown, grayish-brown, tan, dark or blackish in color and have long slender legs and six eyes distributed in three dyads, which are important for identifying the genus. They are nocturnal, mainly insectivorous, sedentary, and not very aggressive (they only bite when threatened). They can hide in logs and under stones, or in dark places with little ventilation and transit, such as behind furniture, frames, and closets, which increases the probability of contact with the household inhabitants ^4^ ^-^ ^6^.

These spiders have two apocrine glands that produce dermonecrotic venom with proteolytic and necrotic action, which is why they are considered of medical importance. The venom is composed of hyaluronidases, esterases, proteases and DNases, mainly phospholipase D (sphingomyelinase D), responsible for the necrotic and hemolytic process. Loxoscelism is the term used to describe the clinical manifestations caused by its toxicity when it is inoculated in humans ^5^ ^,^ ^7^.

Cases of loxoscelism are associated with the distribution of *Loxosceles* spiders. The *Loxosceles* species of greatest medical interest in South America are *L. laeta*, *L. gaucho* and *L. intermedia*; and in North America, *L. reclusa*, *L. deserta*, *L. arizonica* and *L. rufescens* ^8^. The species reported to be associated with attacks on people in Europe are *L. reclusa* and *L. rufescens* ^9^. In Mexico, cases of loxoscelism are erroneously attributed to *L. deserta*, *L. boneti*, *L. arizonica*, and *L. reclusa* ^7^. Globally, most loxoscelism cases have been reported in South American countries and in the United States of America. In Brazil, Chile, Peru, Costa Rica (Central America) and Argentina, loxoscelism is considered a public health problem ^10^ ^,^ ^11^.

In Mexico there is little knowledge about the epidemiology of this disease, as well as an underreporting of *Loxosceles* attacks, largely due to the lack of laboratory methods for diagnosis ^7^. The aim of this article is to report a case of cutaneous loxoscelism associated with *L. yucatana* bites in a resident of Yucatan, Mexico, and to describe part of the epidemiological and public health scenario related to the disease.

## CASE REPORT

A 36-year-old female patient, resident of Merida, Yucatan, Mexico, attended the Merida Hospital Clinic of the Instituto de Seguridad y Servicios Sociales de los Trabajadores del Estado in February 2020, 24 hours after being bitten by a spider at her home. The time of the attack and what the patient was doing at that moment were not specified; however, on the first visit to the clinic, the patient showed photographs of spiders in the peridomicile (backyard) of her home.

**Figure 1:**
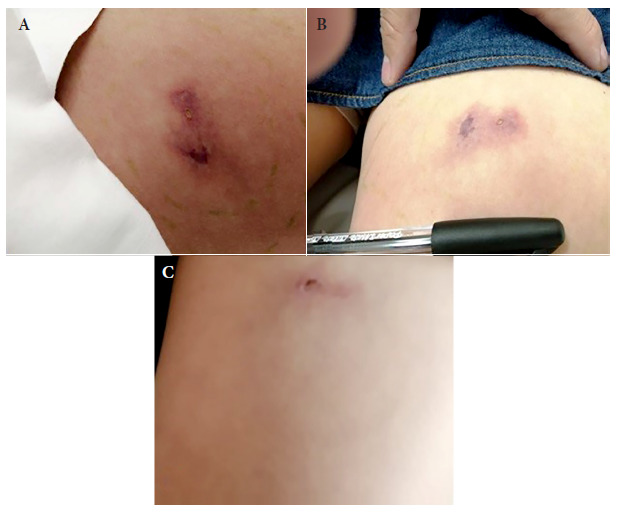
Evolution of the lesions caused by the bite of *Loxosceles yucatana* in a patient from Yucatan, Mexico. A:Appearance of the lesion at the first visit to the clinic (24 h after the bite). B: Appearance of the lesion five days after the bite. C: Appearance of the lesion 28 days after the bite.

At the initial physical examination (first visit), the patient presented a livedoid plaque (diameter was not determined), blister, pain, itching and increased temperature in the middle third of the anterior aspect of the left thigh, referred to as the bite area (Figure 1A). Heart rate (HR), respiratory rate (RR), body temperature (BT) and blood pressure (BP) were stable and within normal parameters. Treatment started at home with an analgesic (diclofenac sodium every 12 h) and a beta-lactam antibiotic (dicloxacillin every 12 h).

The diagnosis of cutaneous loxoscelism caused by *Loxosceles* bite was based on the characteristics of the lesion (vesicle) which were indicative of spider bite, in addition to the symptoms found during the initial physical examination and the photographs shown by the patient. Treatment with polyvalent *loxosceles* antiserum (Reclusmyn®; Laboratorio Sinales; Mexico) was applied intravenously less than 24 h after diagnosis (single dose of 10 ml gauged to 50 ml in saline solution, administered in 30 minutes, according to the manufacturer’s recommendations).

Two days after the attack (second visit), the patient presented mild skin rash and pruritus in the abdomen, therefore, antihistamines (loratadine every 24 h) were added to the treatment. Five days after the attack (third visit), the patient was afebrile, with CR, RR, BT, and BP stable and within normal parameters. The rash and pruritus on the abdomen remained mild, and the lesion presented erythema, increased temperature, flushing, pain on palpation, mild inflammation, and localized induration (1.5 x 1.2 cm in diameter) with a vesicle without devitalized tissue, need for debridement or infection. No signs of necrosis were found (Figure 1B).

Twenty-eight days after the bite, the patient’s evolution was completely satisfactory (Figure 1C) and she was discharged from the hospital. Follow-up was on an outpatient basis because there were no complications.

The patient did not present hematuria during the evolution of the case. Likewise, the blood tests performed after the diagnosis and in the first and second consultations did not show severe hemolysis. All the results obtained from the urine test were within the reference values.

Table 1 shows the main findings in the laboratory tests, as well as the days on which the samples were collected.

**Table 1:** Main findings in the laboratory tests performed on the patient diagnosed with cutaneous loxoscelism in Merida, Yucatan, Mexico.

Laboratory test	Result
Hematology	
First sample	
Hemoglobin	11.6 g/dL
Hematocrit	34.4 %
Eosinophiles	3.5 %
Monocytes	10.3 %
Second sample (48 h after the first sample)	
Hemoglobin	11.9 g/dL
Hematocrit	35.3 %
Eosinophiles	3.2 %
Monocytes	10.4 %
Blood chemistry	
First sample	
Aspartate aminotransferase	36 U/L
Alanine aminotransferase	44 U/L
Second sample	
Aspartate aminotransferase	37 U/L
Alanine aminotransferase	43 U/L

**Figure 2:**
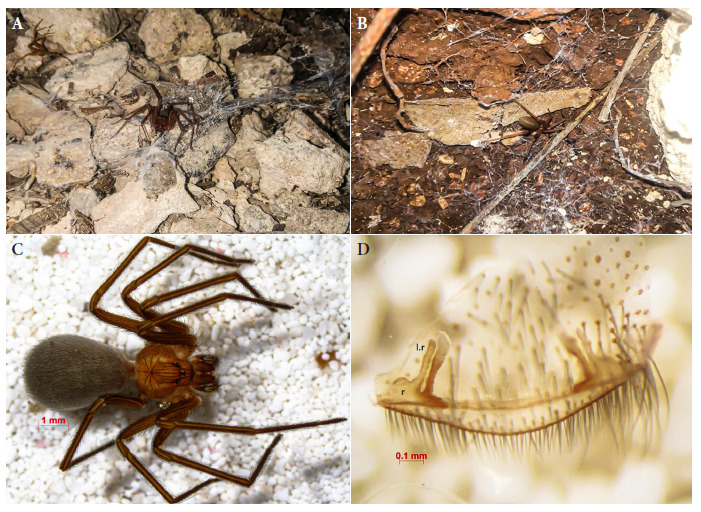
A, B: Spiders collected in the patient’s peridomicile. C: Dorsal view of *Loxosceles yucatana* (female) in which the characteristic violin-shaped spot can be observed. D: Seminal receptacle of *L. yucatana.*

Simultaneously to the first consultation, personnel from the Unidad Colaborativa para Bioensayos Entomológicos (UCBE) of the Campus de Ciencias Biológicas y Agropecuarias, Universidad Autónoma de Yucatán, and the Servicios de Salud de Yucatán, went to the patient’s home to search for spiders and spray insecticide (Ficam W bendiocarb® 80%; Bayer®) with manual compression equipment (IK-Vector Control Super®; Goizper Group®) to deposit a residual dose (1 gr i. a./m^2^) to eliminate possible harmful arachnids. Eleven spiders (3 females and 8 juveniles) were collected from the patient’s peridomicile (Figures 2A, 2B) and preserved in vials with ethanol (70%) for transfer to the UCBE, where the genus was identified. Subsequently, they were sent to the Entomology Laboratory of the Instituto de Diagnóstico y Referencia Epidemiológicos (InDRE) of the Secretaría de Salud (SS), Mexico City (CDMX), in order to identify the species ^12^. All specimens corresponded to the species *L. yucatana* (Figure 2 C, D) and were deposited in the Collection of Arthropods of Medical Importance (CAIM) of the InDRE (registry CAIMAra/oh-01382 to 01392).

## DISCUSSION

Several specimens of *L. yucatana* were collected in the patient’s peridomicile. This finding is significant when talking about cases of loxocelism, because only in less than 5% of the attacks is it possible to identify the aggressor arachnid since it is usually difficult to locate at the moment or in the hours after the attack ^10^ ^,^ ^13^. Likewise, characterization of the species helps to provide adequate treatment within the first hours following the attack, especially because the clinical picture is not distinctive and because it is usually mistaken for other conditions such as cellulitis, erysipelas, arthropod bites, pyoderma gangrenosum, ecthyma and cutaneous anthrax, among others ^14^ ^-^ ^16^.

The first reported case of loxoscelism in Mexico dates back to 1969; since then, many isolated cases have been published ^17^. Recently, cases of systemic loxoscelism in children have been documented ^10^, as well as in a pregnant woman ^18^ and a farmer in Zacatecas ^14^. Nevertheless, *Loxosceles* attacks are still underreported in Mexico, although it is considered that three to five thousand bites per year are caused by these spiders ^7^ ^,^ ^14^ ^,^ ^17^. In this context, Guanajuato, Puebla, Hidalgo, and Veracruz are the states with the highest number of registered attacks ^7^. Particularly, this is the first case of cutaneous loxoscelism in Yucatán described in the literature that is associated with the attack of *L. yucatana*. 

One of the most important aspects of the symptomatology caused by the attack of *Loxosceles* is in which part of the body the bite occurs. The areas where most attacks occur are hands and arms ^15^ ^,^ ^17^, followed by face, pelvis, and thorax ^17^. Although attacks on the legs ^10^ and buttocks ^18^ have also been identified, especially in children who play at ground level or capture the spiders out of curiosity and unawareness of the danger ^10^ ^,^ ^15^. The patient in the present case was attacked on a thigh, an aspect that coincides with most of the attacks reported in the State of Mexico, Sonora, Zacatecas and CDMX ^10^, as well as in children admitted to the Toxicology Information Center of the Department of Continuous Admission and Toxicology of the Mexican Institute of Social Security, CDMX ^15^, and in a patient from Michoacán ^16^.

Greater severity and spread of loxoscelism are associated with attacks in anatomical areas with a predominance of adipose tissue, such as the thighs, as well as with the sex of the spider (female) ^15^ ^-^ ^17^. Likewise, studies report that patients who receive medical attention in the first 36 h after the bite have less severe and less developed lesions ^15^. In this context, the timely medical attention received by the patient in this case (24 h after the bite) helped to prevent progression to a more severe condition.

There are no specific laboratory tests for loxoscelism, so diagnosis is based on the clinical history (anamnesis), the epidemiological presentation, the symptoms and their evolution, and the cutaneous lesion. Results of laboratory tests can provide important information, especially in cases of cutaneous-visceral loxoscelism ^18^; however, some cases have normal values, which does not contribute to the suspicion or diagnosis ^6^.

According to clinical patterns, there are two forms of loxoscelism: local (cutaneous) and cutaneous-visceral (systemic) ^7^ ^,^ ^19^. Cutaneous loxoscelism is the most frequent (84-97% of reported cases) and occurs 6-8 h after the bite. The most common symptoms are fever, pain, pruritus and erythema, vasoconstriction, ischemia, redness, increased temperature, and edema around the bite that causes a necrotic lesion of variable depth (livedoid plaque) that may progress to an ischemic necrotic lesion ^7^ ^,^ ^10^ ^,^ ^19^. Cutaneous-visceral loxoscelism is much less frequent than the cutaneous type, but is more severe. A mortality rate of 15-25% has been reported for this form of loxoscelism, and it increases considerably if patients are not treated immediately. Cutaneous-visceral loxoscelism occurs 12-36 h after the attack; it may cause fever and general malaise, as its characteristic symptoms, and other less frequent ones, such as hemolytic anemia, acute renal failure, disseminated intravascular coagulation and multiorgan failure. The factors associated with this form of loxoscelism are fever and poor overall condition of the patient, as well as the spider bite in the thorax ^20^.

Several approaches have been proposed for the clinical management of both types of loxoscelism, such as: supportive treatment including asepsis of the lesion to avoid infection complications and application of cold compresses, tetanus prophylaxis, use of mild analgesics, corticoids; and in severe cases with systemic involvement, in-hospital management is required, use of antihistamines and prednisone and/or dapsone or other antibiotics, none of them specific. Antiloxosceles antidotes have been reported to have good efficacy from 12 to 36 h after the attack; their use is controversial after this point and their efficacy is doubtful ^21^ ^,^ ^22^.

The polyvalent loxosceles antiserum Reclusmyn® was used in this case, which is an anti-venom for *Loxosceles* bites, derived from horse plasma hyper-immunized with recombinant necrotoxins of *L. reclusa*, *L. boneti* and *L. laeta*. Some observational studies have suggested that its use in the first 36 h after the attack leads to a favorable evolution of patients, reduces the severity of necrotic lesions, and reduces the risk of presenting systemic *loxosceles*, as has been demonstrated in some cases previously reported in Mexico ^23^ ^,^ ^24^. Antivenom for the treatment of *Loxosceles* bites is only available in Brazil, Mexico, and Peru ^25^. It is important to mention that observational studies and published cases show that delay in the use of antivenom between the spider attack, the patient accessing medical care and the start of treatment leads to ineffective administration of antivenom ^24^.

In the presented case, the patient mentioned that the bite occurred in her home; therefore, in order to prevent new attacks, it is important to educate the population about preventive measures such as cleaning and tidying up the home and its surroundings every six months to avoid the accumulation of materials and objects that serve as shelter and protection for spiders ^10^. It is important to highlight the timely diagnosis in this case because it helped to reduce the severity of the lesions, the risk of death, and, secondarily, the costs of medical care ^15^. In Mexico, there are few studies that evaluate the clinical-epidemiological aspects of the different forms of loxoscelism, so there is a need for research to establish the extent and severity of the problem, as well as to develop diagnostic and treatment tools ^3^ ^,^ ^5^ ^,^ ^7^.
