# Supplementary material for: First record of a case of cutaneous loxoscelism caused by violin spider (Loxosceles yucatana) bite in Yucatan, Mexico
Source: Rev Peru Med Exp Salud Publica. 2022 Dec 5;39(4):489–94. doi: 10.17843/rpmesp.2022.394.11047 (PMC11397758; doi:10.17843/rpmesp.2022.394.11047)
Supplement: Translation

# Translation: Primer registro de un caso de loxoscelismo cutáneo ocasionado por mordedura de araña violinista *(Loxosceles yucatana*) en Yucatán, México

**Authors:** Daly Martínez-Ortiz, Marco Torres-Castro, Carlos Arisqueta-Chablé, Beatriz Salceda-Sánchez, Herón Huerta, Jorge Palacio-Vargas, Wilbert Bibiano-Marín, Norma Pavía-Ruz, Pablo Manrique-Saide

**Affiliations:** 1 Servicios de Salud de Yucatán, Mérida, México.; 2 Centro de Investigaciones Regionales “Dr. Hideyo Noguchi”, Universidad Autónoma de Yucatán, Mérida, México.; 3 Unidad Colaborativa para Bioensayos Entomológicos, Campus de Ciencias Biológicas y Agropecuarias, Universidad Autónoma de Yucatán, Mérida, México.; 4 Laboratorio de Entomología, Instituto de Diagnóstico y Referencia Epidemiológicos, Secretaría de Salud, Ciudad de México, México.

**Keywords:** Loxoscelismo cutáneo, *Loxosceles yucatana*, caso clínico, Yucatán, México, arañas

## Abstract

El loxoscelismo es ocasionado cuando el veneno dermonecrótico producido por las arañas del género *Loxosceles,* conocidas como «arañas violinistas», ingresa al organismo de una persona a través de su mordida. En México ocurre un subregistro de los casos de loxoscelismo por la ausencia de pruebas de laboratorio para su diagnóstico y la dificultad del cuadro clínico. El objetivo de este trabajo es describir un caso de loxoscelismo cutáneo ocasionado por mordedura de *Loxosceles yucatana * en un residente de Yucatán, México. El loxoscelismo cutáneo es el tipo más frecuente y menos severo. El presente caso se diagnosticó por medio de la sintomatología registrada en la historia clínica, la lesión inicial y la identificación de arañas *L. yucatana*. Este trabajo representa la primera descripción de un caso de loxoscelismo cutáneo con resolución favorable en Yucatán.

## INTRODUCCIÓN

Las arañas del género *Loxosceles* (L.) pertenecen a la familia Sicariidae, suborden Araneomorphae, orden Araneae. Mundialmente, se conocen 139 especies ^1^, de las cuales se han descrito 40 especies en México (38 nativas y dos introducidas:* L. reclusa *y *L. rufescens*) ^2^ ^,^ ^3^. En el Estado de Yucatán sólo se ha eportado a *L. yucatana,* siendo de hecho, la primera especie de Loxosceles reportada en el país, basándose en ejemplares recolectados en cuevas de este Estado ^3^.

El término Loxosceles (*loxos:* curvas y *kelos:* patas) hace referencia a la posición de sus patas en reposo, ligeramente laterígradas, que les da un aspecto circular o curvo. Son conocidas comúnmente como “arañas marrones”, “arañas del rincón”, “arañas pardas” o “arañas violinistas” porque la mayoría de las especies presentan una característica mancha oscura en el cefalotórax con forma de violín con el mango hacia su extremo posterior ^3^ ^,^ ^4^. Estas arañas miden de 8-13 mm de longitud incluyendo las patas. Son de color marrón, marrón grisáceo, tostado, oscuro o negruzco y tienen patas largas y delgadas y seis ojos distribuidos en tres díadas, importantes para la identificación del género. Son nocturnas, principalmente insectívoras, sedentarias y poco agresivas (sólo muerden al sentirse amenazadas). En las viviendas se alojan en troncos y piedras, o en lugares oscuros con poca ventilación y tránsito como detrás de muebles, cuadros y roperos, lo que aumenta la probabilidad de contacto con los habitantes del hogar ^4^ ^-^ ^6^. 

Estas arañas son consideradas de importancia médica debido a que su veneno es dermonecrótico, con acción proteolítica y necrótica, que es generado en dos glándulas apocrinas. Está compuesto de hialuronidasas, esterasas, proteasas y DNasas, principalmente fosfolipasa D (esfingomielinasa D), responsable del proceso necrótico y hemolítico.Loxoscelismo es el término utilizado para describir las manifestaciones clínicas ocasionadas por su toxicidad cuando se inocula, a través de la mordedura, en personas atacadas ^5^ ^,^ ^7^. 

Los casos de loxoscelismo están asociados con la distribución de las arañas *Loxosceles*. Las especies de *Loxosceles* de mayor interés médico en Suramérica son *L. laeta, L. gauch*o y* L. intermedia*; y en Norteamérica, *L. reclusa, L. deserta, L. arizonica *y *L. rufescens* ^8^. En Europa, las especies reportadas asociadas a ataques en personas son *L. reclusa* y *L. rufescens* ^9^. En México, los cuadros de loxoscelismo se atribuyen erróneamente a *L. deserta, L. boneti, L. arizonica *y *L. reclusa* ^7^. La mayor parte de los casos de loxoscelismo a nivel global se han reportado en países de Sudamérica y en Estados Unidos de América. En Brasil, Chile, Perú, Costa Rica (Centroamérica) y Argentina, el loxoscelismo es considerado un problema de salud pública ^10^ ^,^ ^11^. 

En México existe poco conocimiento sobre la epidemiología de este padecimiento, así como un subregistro de los ataques de Loxosceles, debido en gran parte a que no se cuenta con métodos de laboratorio para el diagnóstico ^7^. El objetivo de este artículo es reportar un caso de loxoscelismo cutáneo asociado a mordedura de *L. yucatana *en un residente de Yucatán, México y describir parte del panorama epidemiológico y de salud pública relacionados con el padecimiento.

## REPORTE DEL CASO

Paciente femenina de 36 años, residente de Mérida, Yucatán, México, acudió a la Clínica Hospital Mérida del Instituto de Seguridad y Servicios Sociales de los Trabajadores del Estado en febrero del 2020, 24 horas después de sufrir una mordedura de araña en su domicilio. La hora del ataque y la actividad que la paciente se encontraba realizando no fueron precisadas; no obstante, en la primera consulta la paciente mostró fotografías de arañas presentes en el peridomicilio (patio) de su vivienda.

Al examen físico inicial (primera consulta), la paciente presentó una placa livedoide (no se determinó su diámetro), flictena, dolor, escozor y aumento de temperatura en el tercio medio de la cara anterior del muslo izquierdo, referida como el área de la mordedura (Figura 1A). El ritmo cardíaco (RC), la frecuencia respiratoria (FR), temperatura corporal (TC) y presión arterial (PA) estaban estables y dentro de los parámetros normales. Se inició tratamiento con manejo domiciliario de analgésico (diclofenaco sódico cada 12 h) y antibiótico betalactámico (dicloxacilina cada 12 h).

**Figura 1:** Evolución de las lesiones ocasionadas por mordedura de Loxosceles yucatana en un paciente de Yucatán, México. A: Aspecto de la lesión en la primera consulta (24 h después de la mordedura). B: Aspecto de la lesión cinco días después de la mordedura. C: Aspecto de la lesión 28 días después de la mordedura.

Se decidió establecer el diagnóstico de loxoscelismo cutáneo ocasionado por mordedura de *Loxosceles* debido a las características de la lesión (vesícula) que eran indicativas de mordedura por araña, además de la sintomatología observada en el examen físico inicial y la sospecha de mordedura por *Loxosceles*, a partir de las fotografías mostradas por la paciente. Se aplicó tratamiento con faboterápico polivalente antiloxosceles (Reclusmyn®; Laboratorio Sinales; México) vía endovenosa menos de 24 h después del diagnóstico (única dosis de 10 ml aforados a 50 ml en solución salina, administrados en 30 minutos, de acuerdo con la recomendación del laboratorio fabricante).

Dos días después del ataque (segunda consulta), la paciente presentó erupción cutánea y prurito leves en el abdomen, por lo que, adicionalmente, se inició tratamiento con antihistamínicos (loratadina cada 24 h). Cinco días después del ataque (tercera consulta), la paciente se encontró afebril, con RC, FR, TC y PA estables y dentro de los parámetros normales. La erupción cutánea y el prurito en el abdomen continuaron leves, y en el sitio de la mordedura se observó eritema, aumento de temperatura, rubor, dolor a la palpación, inflamación ligera e induración localizada (1,5 x 1,2 cm de diámetro) con una vesícula sin tejido desvitalizado, necesidad de desbridamiento o infección. No se observó necrosis Figura1B.

Veintiocho días después de la mordedura, la evolución de la paciente fue totalmente satisfactoria (Figura 1C) por lo que se le otorgó el alta médica. El seguimiento del caso fue ambulatorio debido a que no se presentaron complicaciones.

Durante la evolución del caso la paciente no presentó hematuria. Asimismo, los estudios realizados en sangre, posteriores al diagnóstico y realizados en la primera y segunda consultas, no demostraron hemólisis severa. Todos los resultados obtenidos en el examen general de orina se encontraron dentro de los valores de referencia.

En la Tabla 1 se presentan los principales hallazgos en las pruebas de laboratorio, así como los días en los que se recolectaron las muestras enviadas al laboratorio.

**Tabla 1:** Principales hallazgos en los exámenes de laboratorio realizados a la paciente con diagnóstico de loxoscelismo cutáneo de Mérida, Yucatán, México.

Examen de laboratorio	Resultado
Biometría hemática	
Primera muestra	
Hemoglobina	11,6 g/dL
Hematocrito	34,4 %
Eosinófilos	3,5 %
Monocitos	10,3 %
Segunda muestra (48 h después de la primera muestra)	
Hemoglobina	11,9 g/dL
Hematocrito	35,3 %
Eosinófilos	3,2 %
Monocitos	10,4 %
Química sanguínea	
Primera muestra	
Aspartato aminotransferasa	36 U/L
Alanino aminotransferasa	44 U/L
Segunda muestra	
Aspartato aminotransferasa	37 U/L
Alanino aminotransferasa	43 U/L

Paralelamente a la primera consulta, personal de la Unidad Colaborativa para Bioensayos Entomológicos (UCBE) del Campus de Ciencias Biológicas y Agropecuarias, Universidad Autónoma de Yucatán, y de los Servicios de Salud de Yucatán, acudieron al domicilio de la paciente para realizar una búsqueda de arañas y un rociado de insecticida (Ficam W® bendiocarb® 80%; Bayer®) con equipo de compresión manual (IK-Vector Control Super®; Goizper Group®) para depositar una dosis residual (1 gr i.a./m^2^) que elimine posibles arácnidos nocivos. En el peridomicilio de la paciente se recolectaron 11 arañas (3 hembras y 8 juveniles) (Figura 2 A, B) que se conservaron en frascos con etanol (70%) para su traslado a la UCBE donde se realizó la identificación del género. Posteriormente, fueron enviadas al Laboratorio de Entomología del Instituto de Diagnóstico y Referencia Epidemiológicos (InDRE) de la Secretaría de Salud (SS), Ciudad de México (CDMX), para iden tificar la especie ^12^. Todos los ejemplares correspondieron a la especie* L. yucatana* (Figura 2C, D) y fueron depositaron en la Colección de Artrópodos de Importancia Médica (CAIM) del InDRE (registro CAIMAra/oh-01382 al 01392).

**Figura 2:** A, B: Arañas recolectadas en el peridomicilio de la paciente. C: Vista dorsal de* Loxosceles yucatana *(hembra) en la que puede observarse la mancha característica en forma de violín. D: Receptáculo seminales de *L. yucatana.*

## DISCUSIÓN

En el peridomicilio de la paciente se lograron recolectar e identificar varios ejemplares de *L. yucatana*. Este hallazgo es significativo al hablar de casos de loxocelismo, debido a que en menos del 5% de los ataques se consigue identificar al arácnido agresor ya que es difícil encontrarlo al momento o en las horas posteriores al ataque ^10^ ^,^ ^13^. De igual manera, la caracterización de la especie agresora ayuda a otorgar el tratamiento correcto en las primeras horas de evolución, sobre todo, porque el cuadro clínico no es característico y porque suele confundirse con otras patologías como celulitis, erisipela, mordeduras de artrópodos, pioderma gangrenoso, ectima y ántrax cutáneo, entre otras ^14^ ^-^ ^16^.

El primer caso registrado de loxoscelismo en México data de 1969, a partir de entonces se han publicado numerosos casos aislados ^17^. Recientemente, se han documentado casos de loxoscelismo sistémico en niños ^10^, una mujer embarazada ^18^ y en un campesino de Zacatecas ^14^. A pesar de esto, aún existe un subregistro de los ataques de Loxosceles en México, aunque se considera que ocurren de tres a cinco mil mordeduras al año provocadas por estas arañas ^7^ ^,^ ^14^ ^,^ ^17^. En este contexto, los Estados que presentan la mayor cantidad de ataques registrados son Guanajuato, Puebla, Hidalgo y Veracruz^ (^ ^7^. Particularmente, en Yucatán, el presente es el primer caso de loxoscelismo cutáneo descrito en la bibliografía asociado al ataque de *L. yucatana*.

Uno de los aspectos más importantes en la sintomatología generada por el ataque de Loxosceles, es el área corporal donde se produce la mordedura. Se ha observado que las áreas más atacadas son manos y brazos ^15^ ^,^ ^17^, seguidos de cara, pelvis y tórax ^17^. Aunque también se han identificado ataques en pierna^s (^ ^10^ y glúteos^ (^ ^18^, sobre todo en niños que juegan a nivel del suelo o capturan a las arañas por curiosidad y desconocimiento de su peligro ^10^ ^,^ ^15^. La paciente del presente caso fue atacada en un muslo, aspecto que coincide con la mayor parte de los ataques registrados en el Estado de México, Sonora, Zacatecas y CDMX ^10^, así como en niños ingresados en el Centro de Información Toxicológica del Departamento de Admisión Continua y Toxicología del Instituto Mexicano del Seguro Social, CDMX ^15^, y en un paciente de Michoacán ^16^. 

La mayor severidad y extensión del cuadro de loxoscelismo están asociados con ataques en áreas anatómicas con predominio de tejido adiposo, como los muslos, así como con el sexo de la araña (hembra) ^15^ ^-^ ^17^. Asimismo, está descrito que aquellos pacientes que reciben atención médica en las primeras 36 h después de la mordedura presentan una menor severidad y desarrollo de las lesiones^ (^ ^15^. En este contexto, la atención médica oportuna que recibió la paciente en el caso presentado (24 h después de la mordedura) ayudó a que el cuadro no evolucione a una mayor severidad.

No existen pruebas específicas de laboratorio para el diagnóstico de loxoscelismo, por lo que éste se basa en la historia clínica (anamnesis), la presentación epidemiológica, los síntomas y su evolución, y la lesión cutánea. Los resultados de los estudios de gabinete pueden arrojar información para sospechar del evento clínico, sobre todo en los casos de loxoscelismo cutáneo-visceral ^18^; sin embargo, algunos casos presentan valores normales, lo cual no contribuye con la sospecha o el diagnóstico^ (^ ^6^. 

De acuerdo con los patrones clínicos, existen dos formas de loxoscelismo: local (cutáneo) y cutáneo-visceral (sistémico) ^7^ ^,^ ^19^. El loxoscelismo cutáneo es el más frecuente (84-97% de los casos reportados) y se presenta de 6-8 h después de la mordedura. Los síntomas más comunes son fiebre, dolor, prurito y eritema, vasoconstricción, isquemia, enrojecimiento, aumento de temperatura y edema alrededor de la mordedura que ocasionan una lesión necrótica de profundidad variable (placa livedoide) que puede progresar a lesión necrótica isquémica ^7^ ^,^ ^10^ ^,^ ^19^. El loxoscelismo cutáneo-visceral es mucho menos frecuente que el tipo cutáneo, pero es más severo. Genera entre un 15 y 25% de mortalidad que aumenta considerablemente si los pacientes no son atendidos inmediatamente. Ocurre de 12-36 h después del ataque; puede ocasionar fiebre y malestar general, como síntomas característicos, y otros menos frecuentes, como anemia hemolítica, insuficiencia renal aguda, coagulación intravascular diseminada y falla multiorgánica. Los factores asociados a la ocurrencia de esta forma de loxoscelismo son fiebre y mal estado general del paciente, así como la mordedura de araña localizada en tórax ^20^.

Se han propuesto numerosos abordajes para el manejo clínico de ambos tipos de loxoscelismo como: tratamiento de sostén que incluye asepsia de la lesión para evitar complicaciones de infección y aplicación de compresas frías, profilaxis antitetánica, uso de analgésicos leves, corticoides; y en los casos graves con compromiso sistémico, se requiere manejo intrahospitalario, uso de antihistamínicos y de prednisona y/o dapsona u otros antibióticos, ninguno de ellos específico. En relación con el uso de los antídotos antiloxosceles, se ha descrito buena eficacia de 12 a 36 h después delataque; transcurrido este tiempo, su uso es controversial y su eficacia es dudosa ^21^ ^,^ ^22^.

En el presente caso se utilizó el foboterápico polivalente antiloxosceles Reclusmyn® que es un anti veneno para mordedura por *Loxosceles*, derivado del plasma de caballo hiperinmunizado con necrotoxinas recombinantes de *L. reclusa, L. boneti* y *L. laeta*. Algunos estudios observacionales han sugerido que su aplicación en las primeras 36 h posteriores al ataque, permite la evolución favorable de los pacientes, reduce la severidad de las lesiones necróticas y disminuye el riesgo de presentar loxoscelismo sistémico, como se ha demostrado en algunos casos descritos previamente en México ^23^ ^,^ ^24^. El anti veneno para el tratamiento de accidentes con arañas *Loxosceles* únicamente está disponible en Brasil, México y Perú ^25^. Es importante mencionar que, los estudios observacionales y los casos publicados demuestran que, un retraso en la aplicación del anti veneno entre el ataque de la araña, la presentación del paciente a consulta y el inicio del tratamiento, conllevan a una administración inefectiva del anti veneno ^24^. 

La paciente del caso que presentamos mencionó que la mordedura ocurrió en su hogar; por lo tanto, para prevenir ataques nuevos, es importante educar a la población sobre medidas de prevención como limpiar y ordenar cada seis meses las viviendas y sus alrededores para evitar la acumulación de materiales y objetos que sirven como resguardo y protección a las arañas^ (^ ^10^. Es importante destacar el diagnóstico oportuno en el presente caso debido a que ayudó a disminuir la gravedad de las lesiones, el riesgo de muerte, y, secundariamente, los costos de atención médica^ (^ ^15^. En México, son escasos los estudios que evalúan los aspectos clínico-epidemiológicos de los tipos de loxoscelismo, por lo que existe la necesidad de realizar investigaciones que establezcan la dimensión y gravedad del problema, o generen herramientas de diagnóstico y tratamiento^ (^ ^3^ ^,^ ^5^ ^,^ ^7^.
